# Supplementary material for: Chromium Complexes Supported by Salen-Type Ligands for the Synthesis of Polyesters, Polycarbonates, and Their Copolymers through Chemoselective Catalysis
Source: Int J Mol Sci. 2023 Apr 21;24(8):7642. doi: 10.3390/ijms24087642 (PMC10144741; doi:10.3390/ijms24087642)
Supplement: Supplementary file 1 [file ijms-24-07642-s001.zip › ijms-2318450-supplementary.pdf]

<u>Supporting Information</u>

1. Synthesis of Complexes 1-3 2

Synthesis of SalenCrCl complex ***Cr1*** 3

Synthesis of SalalenCrCl complex ***2*** 3

Synthesis of SalanCrCl complex ***3*** 4

2. Characterization of Complexes 1-3 4

MALDI-ToF mass spectrometry 4

UV-visible spectroscopy 6

FT-IR spectroscopy 7

3.1 Ring-opening Copolymerization of phthalic anhydride (PA) and cyclohexene oxide (CHO) .. 8

3.2 Ring-opening Copolymerization of phthalic anhydride (PA) and limonene oxide (LO) 9

3.3 Ring-opening Copolymerization of propylene oxide (PO) and maleic anhydride 10

3.4 Terpolymerization of maleic anhydride (PA), propylene oxide (CHO) and glycolide (GL) .. 11

3.5 Depolymerization of poly(cyclohexene carbonate) 12

4 Characterization of Polymers 13

NMR characterization 13

Gel permeation chromatography analysis (GPC) 16

Thermal gravimetric analysis (TGA) 19

Differential scanning calorimetry (DSC) 21

5 References 23

General considerations

All manipulations of air- and/or water-sensitive compounds were carried out using standardSchlenk or glovebox techniques under a N2 atmosphere. Glassware and vials used in the polymerizations were dried in an oven at 120 °C overnight and exposed to vacuum-nitrogen cycles thrice. All hydrocarbon solvents were distilled over sodium benzophenone The aluminum precursor AlMe3 and CrCl2 was purchased from Aldrich and used as received. Monomers was purchased from Aldrich. All other chemicals were commercially available and used as received unless otherwise stated. Deuterated solvents were purchased from Cambridge Isotope Laboratories, Inc., degassed, and dried over activated 3-Å molecular sieves prior to use.

Instruments and Measurements

The NMR spectra were recorded on Bruker Avance 400 and 600 MHz spectrometers at 25 °C, unless otherwise stated. Chemical shifts (δ) are listed as parts per million and coupling constants (J) in hertz. ^{1}H NMR spectra are referenced using the residual solvent peak at δ = 7.16 for C_6D_6 and δ = 7.27 for $CDCl_3$. ^{13}C NMR spectra are referenced using the residual solvent peak at δ = 128.39 for C6D6 and δ = 77.23 for $CDCl_3$.

The number-average molecular weights (M_n) and molecular weight distributions of polymers (dispersity, Đ) were evaluated by size exclusion chromatography (SEC), using Agilent 1260 Infinity Series GPC (ResiPore 3 µm, 300 ×7.5 mm, 1.0 mL min-1, UV (250 nm) and refractive index (RI, PLGPC 220) detector. All measurements were performed with THF as the eluent at a flow rate of 1.0 mL/min at 35°C. Monodisperse poly(styrene) polymers were used as calibration standards

MALDI-ToF-MS analysis was performed on a Waters Maldi Micro MX equipped with a 337 nm nitrogen laser. An acceleration voltage of 25 kV was applied. The polymer sample was dissolved in THF with Milli-Q water containing 0.1% formic acid at a concentration of 0.8 mg mL^{-1}. The matrix used was 2,5-dihydroxybenzoic acid (DHBA) (Pierce) and was dissolved in THF at a concentration of 30 mg mL^{-1}.

1. Synthesis of Complexes 1-3

*Synthesis of SalenCrCl complex **1***

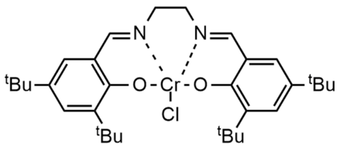

SalenCrCl - 1

SalenCrCl complex **1** was prepared according to a published procedure[1]. In a 100 mL flask equipped with a magnetic stirrer, 200 mg ($4.0x10^{-4}$ mol) of salen ligand and 55 mg ($4.5x10^{-4}$ mol) of chromium (II) chloride $CrCl_2$ were added. The two solids were dissolved in 40 mL of THF and the solution was stirred at room temperature in a nitrogen atmosphere for 24 hours. The solution turned dark red / brown. The following day the solution was exposed to the air for two hours to allow the oxidation of chromium (II) to chromium (III) and was stirred for a further 24 hours. 50 mL of diethyl ether was added to the flask and the solution was extracted with a saturated solution of NH_4Cl (3x100mL) and a saturated solution of NaCl (3x100mL). The organic phase was dried with Na_2SO_4, filtered and concentrated. The product is a dark orange powder. Yield: 81%.

Characterization of salenCrCl complex **1** was done by means of IR and UV-vis spectroscopy, MALDI-ToF mass spectrometry and Evans NMR technique. IR (KBr, cm^{-1}): 2954, 2905, 1630 (C=N), 1531, 1460, 1436, 1412, 1388, 1361, 1333, 1318, 1272, 1256, 1233, 1201, 1167, 1087, 1024, 965, 911, 872, 857, 832, 809, 784, 746, 701, 539 (Cr-N), 486 (Cr-O). UV−vis (CH_3CN, 0.08 mM, 25 °C, ε = $L \cdot mol^{-1} \cdot cm^{-1}$): 285 nm, 345 nm, 432 nm. MS (MALDI-ToF) m/z: 542,293 ($Cr[Salen]^+$), 559,260 (Cr[Salen]+ H_2O). Magnetic moment (298 K) μ_{eff} = 3,15 μ_B.

*Synthesis of SalalenCrCl complex **2***

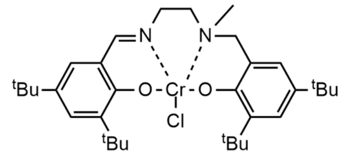

SalalenCrCl - 2

The SalalenCrCl **2** complex was prepared according to the same procedure for complex **1**. The product is a brown-green powder. Yield: 72%.

Characterization of salalenCrCl complex **2** was done by means of IR and UV-vis spectroscopy, MALDI-ToF mass spectrometry and Evans NMR technique. IR (KBr, cm^{-1}): 2954, 2904, 1625 (C=N), 1532, 1475, 1443, 1414, 1391, 1361, 1312, 1274, 1257, 1238, 1202, 1169, 1120, 1077, 1025, 875, 839, 811, 784, 745, 618, 547 (Cr-N), 490 (Cr-O). UV−vis (CH_3CN, 0.08 mM, 25 °C, ε = $L \cdot mol^{-1} \cdot cm^{-1}$): 232 nm, 282 nm, 416 nm. MS (MALDI-ToF) m/z: 558,325 ($Cr[Salen]^+$), 574,320 (Cr[Salen]+ H_2O), 590,315 (Cr[Salen]+ O_2). Magnetic moment (298 K) μ_{eff} = 3,06 μ_B.

*Synthesis of SalanCrCl complex **3***

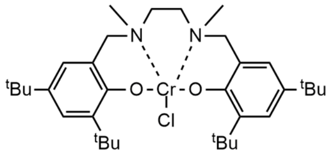

SalanCrCl - 3

The SalanCrCl **3** complex was prepared according to the same procedure for complex **1**. The product is a petrol green powder. Yield: 67%.

Characterization of salanCrCl complex **3** was done by means of IR and UV-vis spectroscopy, MALDI-ToF mass spectrometry and Evans NMR technique. IR (KBr, cm^{-1}): 2954, 2903, 1543, 1470, 1442, 1423, 1413, 1390, 1361, 1305, 1274, 1251, 1238, 1203, 1167, 1133, 1054, 1018, 966, 914, 876, 838, 808, 764, 746, 684, 622, 548 (Cr-N), 491 (Cr-O). UV−vis (CH_3CN, 0.08 mM, 25 °C, $\varepsilon = L \cdot mol^{-1} \cdot cm^{-1}$): 246 nm, 289 nm, 569 nm. MS (MALDI-ToF) m/z: 574,356 ($Cr[Salen]^+$), 592,366 (Cr[Salen]+ H_2O). Magnetic moment (298 K) μ_{eff} = 3,08 μ_B.

2. Characterization of Complexes 1-3

MALDI-ToF mass spectrometry

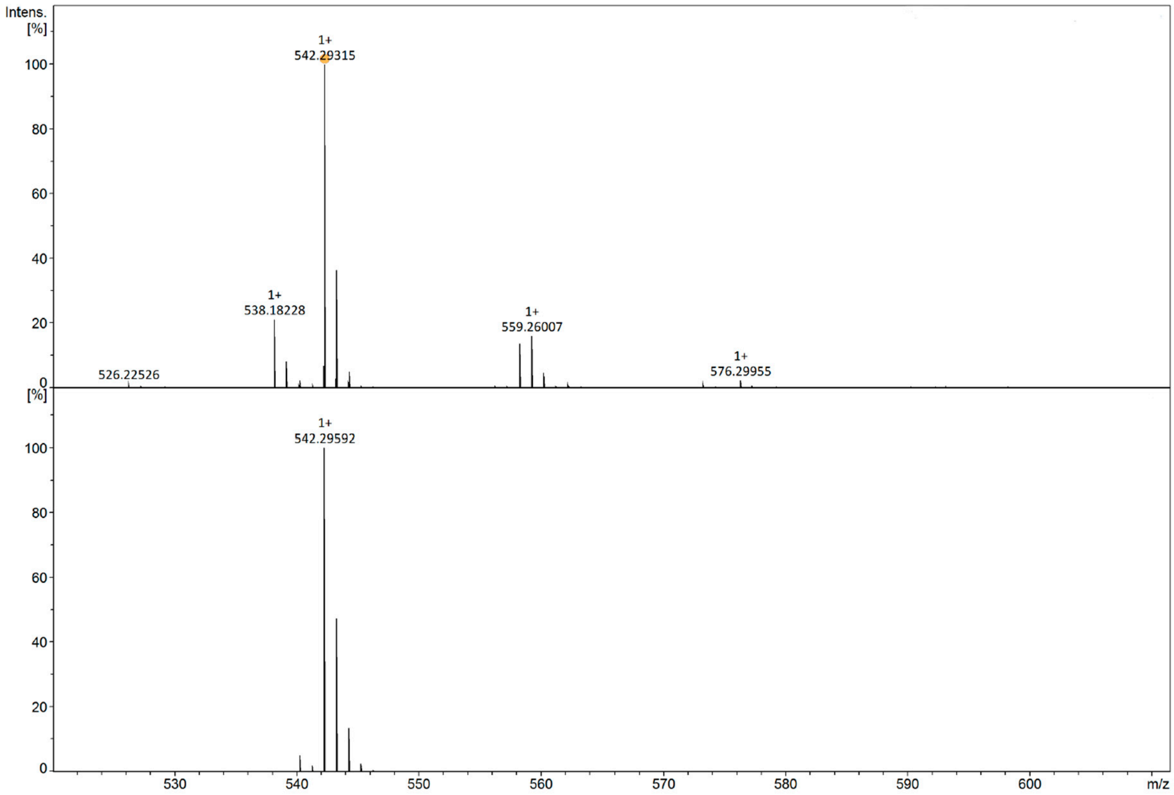

Figure S1. MALDI-ToF mass spectrum of complex **1**

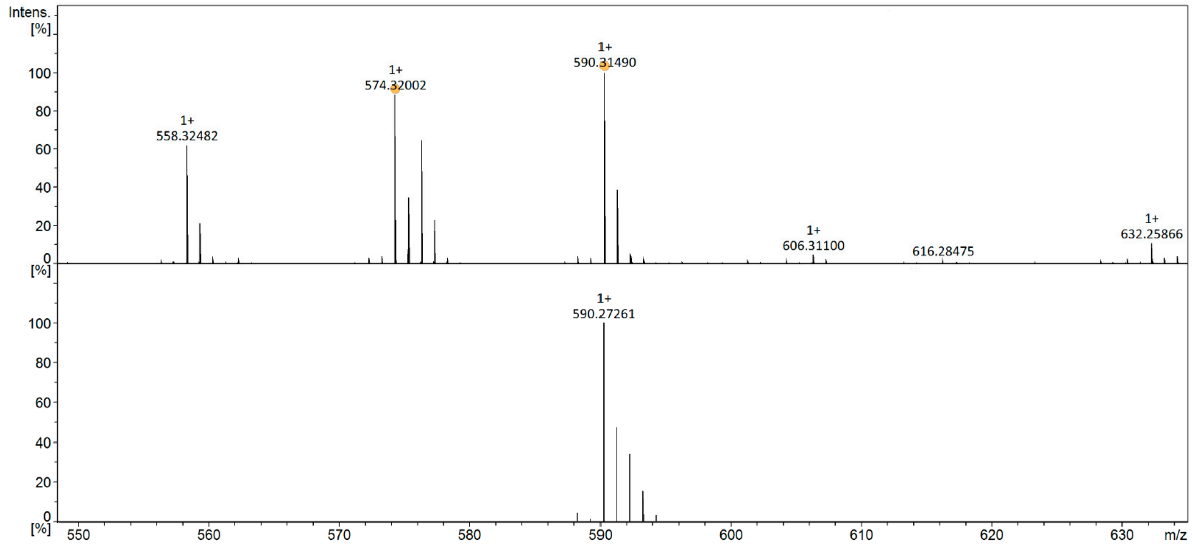

Figure S2. MALDI-ToF mass spectrum of complex **2**

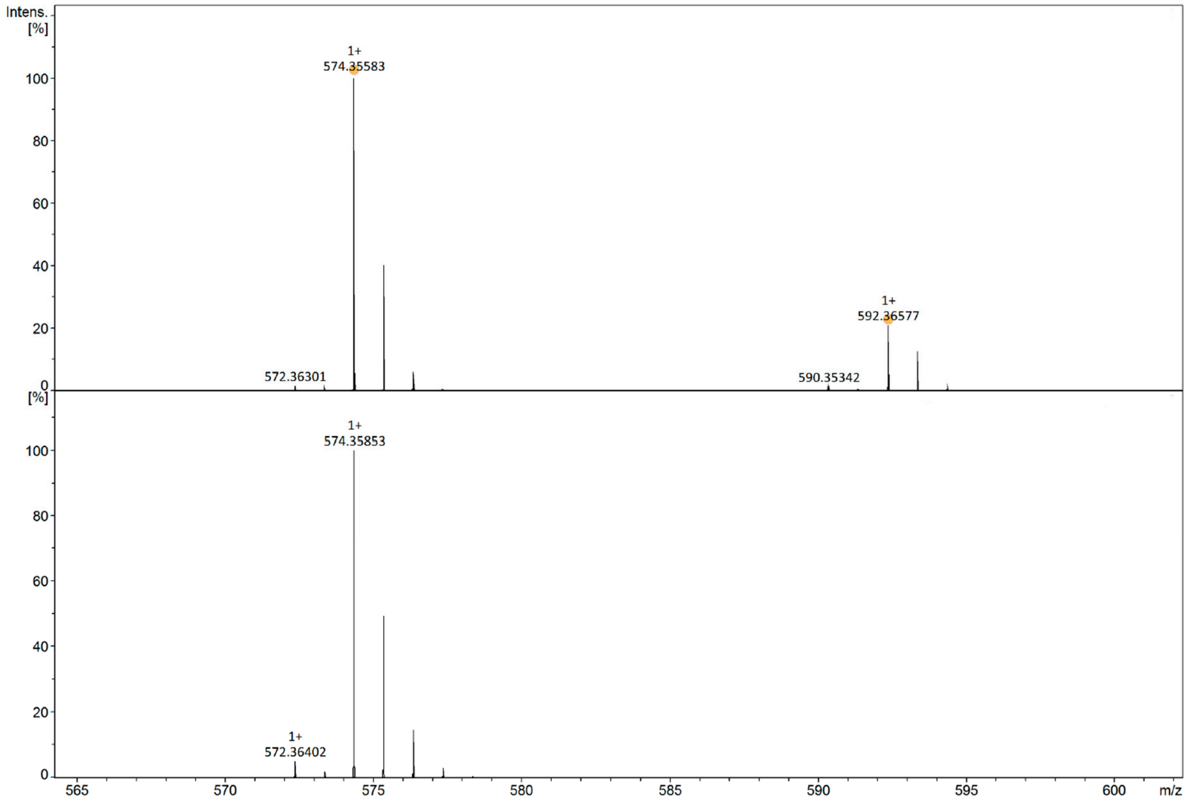

Figure S3. MALDI-ToF mass spectrum of complex **3**

UV-visible spectroscopy

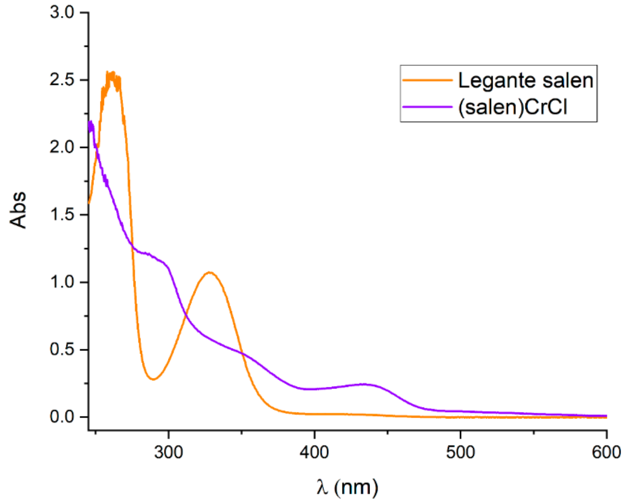

Figure S4. Electronic absorption spectra of salen ligand and complex **1** in acetonitrile

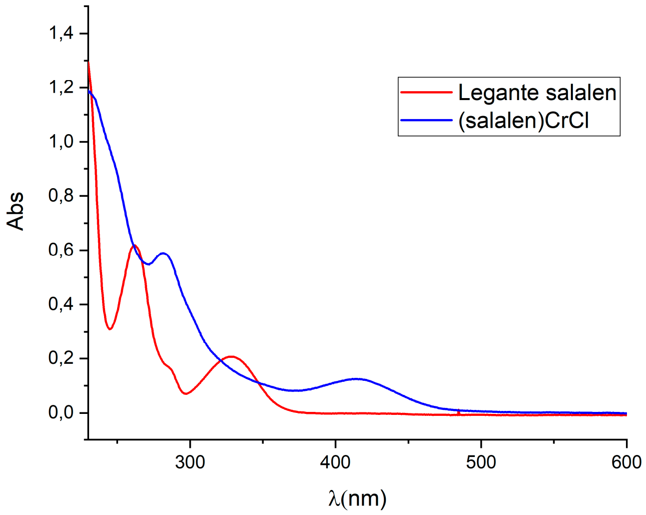

Figure S5. Electronic absorption spectra of salalen ligand and complex **2** in acetonitrile

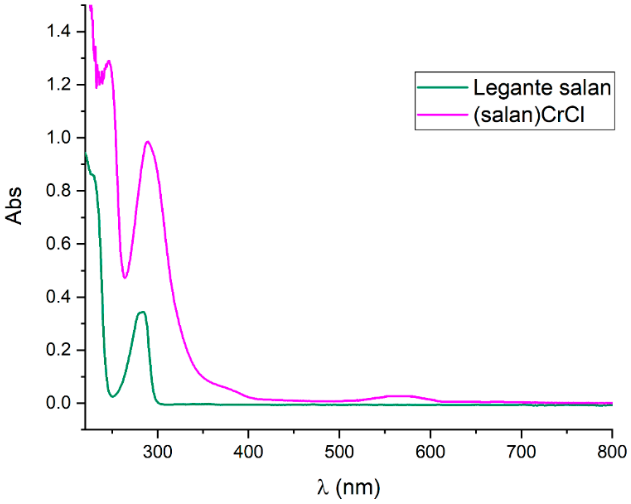

Figure S6. Electronic absorption spectra of salan ligand and complex **3** in acetonitrile

FT-IR spectroscopy

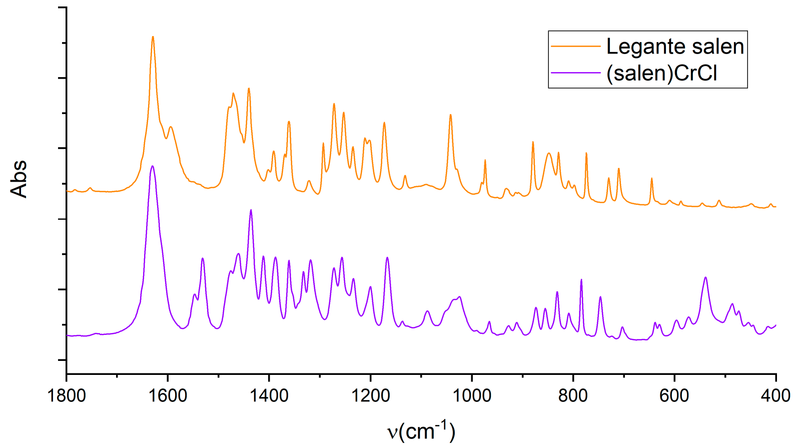

Figure S7. FT-IR spectra of salen ligand and of the corresponding complex **1**

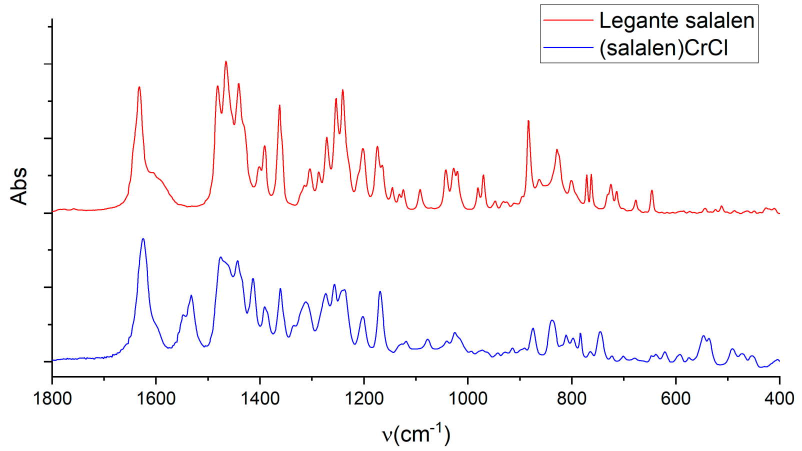

Figure S8. FT-IR spectra of salalen ligand and of the corresponding complex **2**

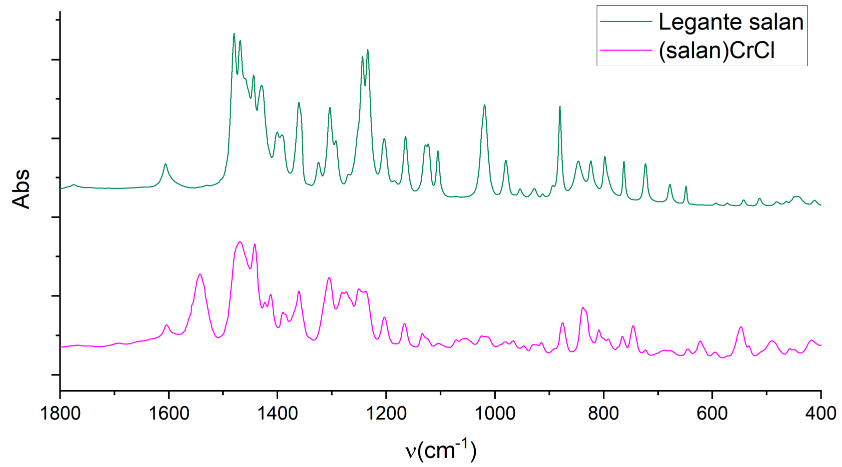

Figure S9. FT-IR spectra of salan ligand and of the corresponding complex **3**

3.1 Ring-opening Copolymerization of phthalic anhydride (PA) and cyclohexene oxide (CHO)

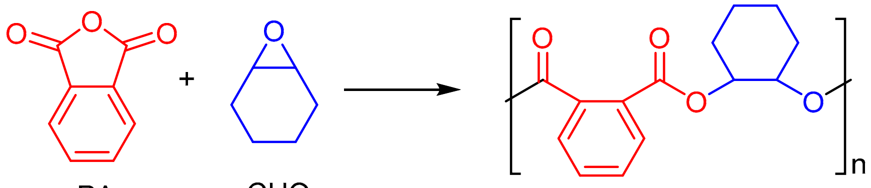

General procedure: Phthalic anhydride (PA, 1 mmol, 100 eq), co-catalyst (PPNCl, 10 µmol, 1 eq), metal complex (complexes **1-4**, 10 µmol, 1 eq) and cyclohexene oxide (CHO, 0.01 mol, 1000 eq) were added to a 10 mL Schlenk tube equipped with a magnetic stirrer. The system was stirred in an oil bath at 70 °C and at different times of 15, 30 and 60 minutes, a small sample of the crude reaction mixture was used to calculate the conversion by ^{1}H NMR spectroscopy. At the end of the polymerization, a minimum amount of CH_2Cl_2 was added and the polymer was coagulated in methanol. The obtained polymer was recovered by filtration and dried in the vacuum oven overnight.

Table S1. PA/CHO copolymerization promoted by complexes **1-4**

Entry	Cat	Co-cat	Time (min)	Conv. (%)	M_n (Kg/mol)	$M_{n\,(teo)}$ (Kg/mol)	Đ
1	1	PPNCl	15	31			
			30	93	0.95	22.9	3.30
2	2	PPNCl	15	26			
			30	46			
			60	100	4.84	24.6	1.30
3	3	PPNCl	15	12			
			30	31			
			60	87	1.23	21.4	3.17
4	4	PPNCl	15	47			
			30	100	3.70	24.6	1.19

General conditions: complexes 1-4 (10 µmol, 1 eq), PPNCl (10 µmol, 1 eq), PA (1 mmol, 100 eq), CHO (1 mL, 1000 eq), T= 70°C, neat.

3.2 Ring-opening Copolymerization of phthalic anhydride (PA) and limonene oxide (LO)

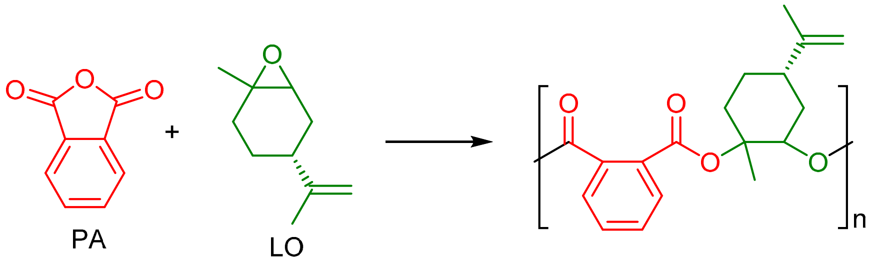

General procedure: Phthalic anhydride (PA, 1 mmol, 100 eq), co-catalyst (PPNCl, 10 µmol, 1 eq), metal complex (complexes **1-4**, 10 µmol, 1 eq) and limonene oxide (LO, 5 mmol, 500 eq) were added to a 10 mL Schlenk tube equipped with a magnetic stirrer. The system was stirred in an oil bath at 130 °C for 15 minutes. A small sample of the crude reaction mixture was used to calculate the conversion by ^{1}H NMR spectroscopy. At the end of the polymerization, a minimum amount of CH_2Cl_2 was added and the polymer was coagulated in methanol. The obtained polymer was recovered by filtration and dried in the vacuum oven overnight.

Table S2. PA/LO copolymerization promoted by complexes **1-4**

Entry	Cat	Co-cat	Conv. (%)	M_n (Kg/mol)	$M_{n\ (teo)}$ (Kg/mol)	Đ
1	1	PPNCl	63	3.76	18.9	1.24
2	2	PPNCl	49	4.37	14.7	1.24
3	3	PPNCl	43	1.03	12.9	1.47
4	4	PPNCl	100	3.52	30.0	1.26

General conditions: complexes 1-4 (10 µmol, 1 eq), PPNCl (10 µmol, 1 eq), PA (1 mmol, 100 eq), LO (0,8 mL, 500 eq), T= 130°C, time= 15 min, neat.

3.3 Ring-opening Copolymerization of cyclohexene oxide (CHO) and CO_2

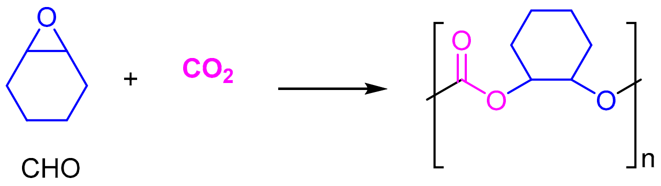

General procedure: In a glovebox, metal complex (complexes **1-4**, 40 µmol, 1 eq) and co-catalyst (PPNCl, 40 µmol, 1 eq) were dissolved in the cyclohexene oxide (CHO, 0.04 mol, 1000 eq) and then transferred into an autoclave. The autoclave was pressurized at 13 bars of pressure of CO_2, and the mixture was allowed to stir at 70°C for 3 hours. After the prescribed time, the reaction mixture was quenched by immersing the autoclave in an ice bath, opened at air and a small sample of the crude reaction mixture was used to calculate the conversion by ^{1}H NMR spectroscopy.

Table S3. CHO/CO_2 copolymerization promoted by complexes **1-4**

Entry	Cat	Co-cat	Conv. (%)	M_n (Kg/mol)	$M_{n\,(teo)}$ (Kg/mol)	Đ
1	**1**	PPNCl	14	-	19.9	
2	**2**	PPNCl	36	7.44	51.2	1.22
3	**3**	PPNCl	27	6.22	38.4	1.25
4	**4**	PPNCl	47	9.61	66.8	1.17

General conditions: complexes 1-4 (40 µmol, 1 eq), PPNCl (40 µmol, 1 eq), CHO (4 mL, 1000 eq), P_{CO2}= 13 bar, T= 70°C, time= 3 h, neat.

3.4 Terpolymerization of phthalic anhydride (PA), cyclohexene oxide (CHO) and CO_2

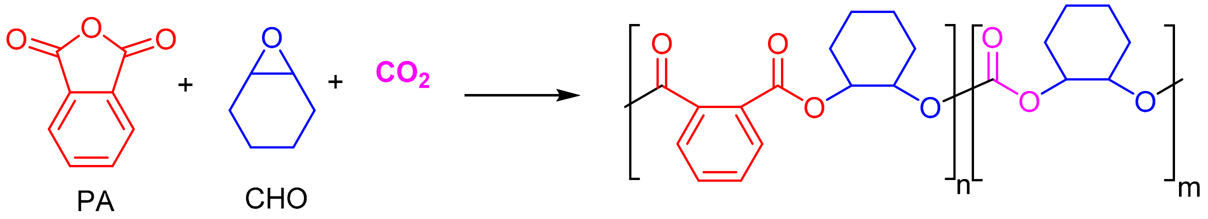

General procedure: In a glovebox, phthalic anhydride (PA, 4 mmol, 100 eq), metal complex (complexes **1-4**, 40 µmol, 1 eq), co-catalyst (PPNCl, 40 µmol, 1 eq) and cyclohexene oxide (CHO, 0.04 mol, 1000 eq) were added to an autoclave. The autoclave was pressurized at 13 bars of pressure of CO_2, and the mixture was allowed to stir at 70°C for 3 hours. After the prescribed time, the reaction mixture was quenched by immersing the autoclave in an ice bath, opened at air and a small sample of the crude reaction mixture was used to calculate the conversion by ^{1}H NMR spectroscopy.

Table S4. PA/CHO/CO_2 terpolymerization promoted by complexes **1-4**

Entry	Cat	Co-cat	n:m	M_n (Kg/mol)	$M_{n\ (teo)}$ (Kg/mol)	Đ
**1**	1	PPNCl	47:53	6.26	36.4	1.23
**2**	2	PPNCl	37:63	8.40	38.6	1.23
**3**	3	PPNCl	48:52	7.21	36.4	1.25
**4**	4	PPNCl	28:72	8.31	48.2	1.16

General conditions: complexes 1-4 (40 µmol, 1 eq), PPNCl (40 µmol, 1 eq), PA (4 mmol, 100 eq), CHO (4 mL, 1000 eq), P_{CO2}= 13 bar, T= 70°C, time= 3 h, neat.

3.5 Depolymerization of poly(cyclohexene carbonate)

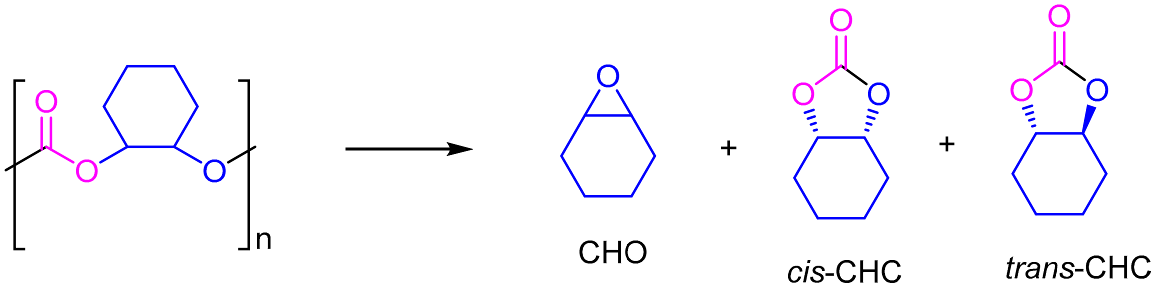

General procedure: In a glovebox, polycarbonate (PCHC, 1.5 mmol, 500 eq to carbonate unit) and co-catalyst (PPNCl, 3 µmol, 1 eq) were added to 10 mL flask. Metal complex (complexes **1-3**, 3 µmol, 1 eq) was dissolved in 1 mL CH_2Cl_2 and transfer in the flask. Then the solvent was removed under reduced pressure and closed in static vacuum. The residue was allowed to 190 °C oil bath. After the prescribed time, the reaction mixture was cooled to room temperature and a small sample of the crude reaction mixture was used to calculate the conversion and the degraded products by ^{1}H NMR spectroscopy.[2]

Table S5. Depolymerization of poly(cyclohexene carbonate), PCHC, using chromium complexes **1-3**

Entry	Cat	time (min)	Conv (%)	CHO (%)	*cis*-CHC (%)	*trans*-CHC (%)
1	1	10	94	97	2	1
2	1	5	75	96	2	2
3	2	5	56	95	2	3
4	3	5	12	95	3	2
5[a]	1	5	23	19	15	2

General conditions: Reactions were performed in bulk at 190 °C under static vacuum condition in a closed 10 mL flask with complexes **1-3** (3 µmol, 1 eq), PPNCl (3 µmol, 1 eq), PCHC (1.5 mmol, 500 eq) refers to repeated carbonate unit. [a]Using terpolymer PA/CHO/CO_2 (1.5 mmol, 500 eq) refers to repeated carbonate unit.

4 Characterization of Polymers

NMR characterization

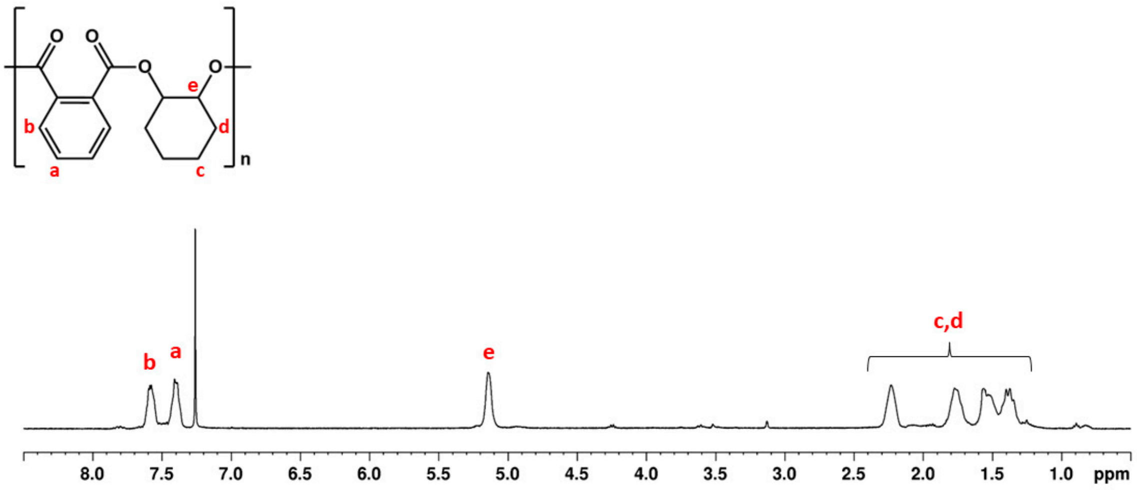

Figure S10. ^{1}H NMR spectrum of polyester poly(PA-*alt*-CHO) in $CDCl_3$ (400 MHz, 298 K)

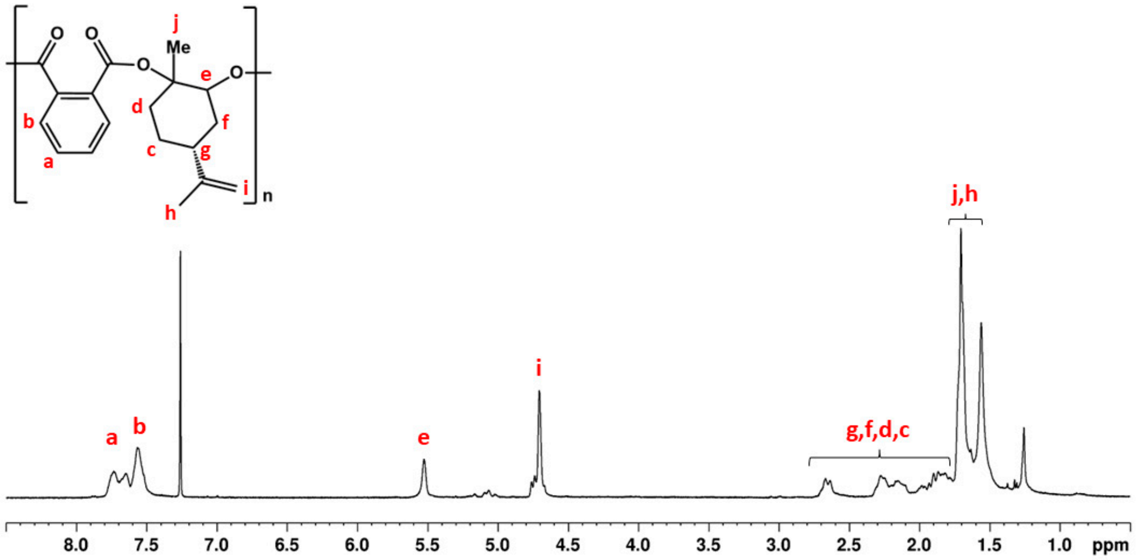

Figure S11. ^{1}H NMR spectrum of polyester poly(PA-*alt*-LO) in $CDCl_3$ (400 MHz, 298 K)

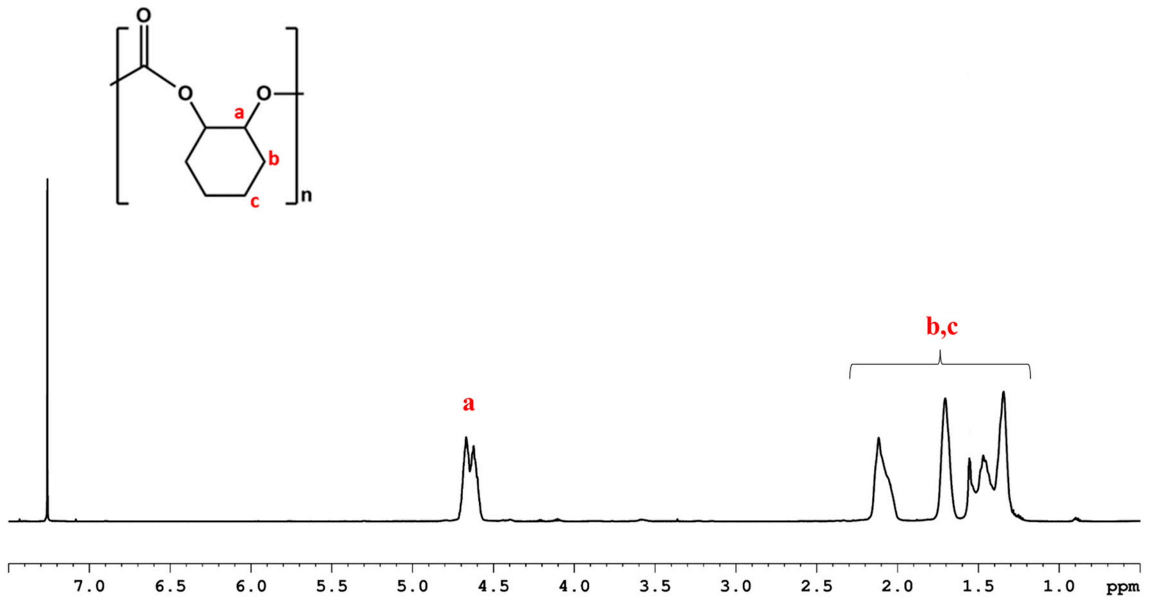

Figure S12. ^{1}H NMR spectrum of poly(cyclohexene carbonate) in $CDCl_3$ (600 MHz, 298 K)

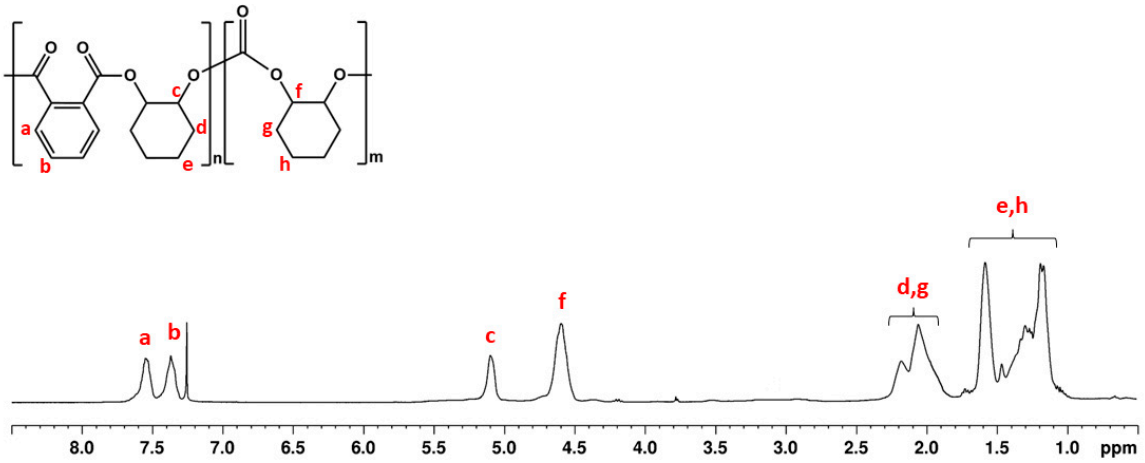

Figure S13. ^{1}H NMR spectrum of poly(ester-*block*-carbonate) from mixture of PA, CHO and CO_2 in $CDCl_3$ (400 MHz, 298 K)

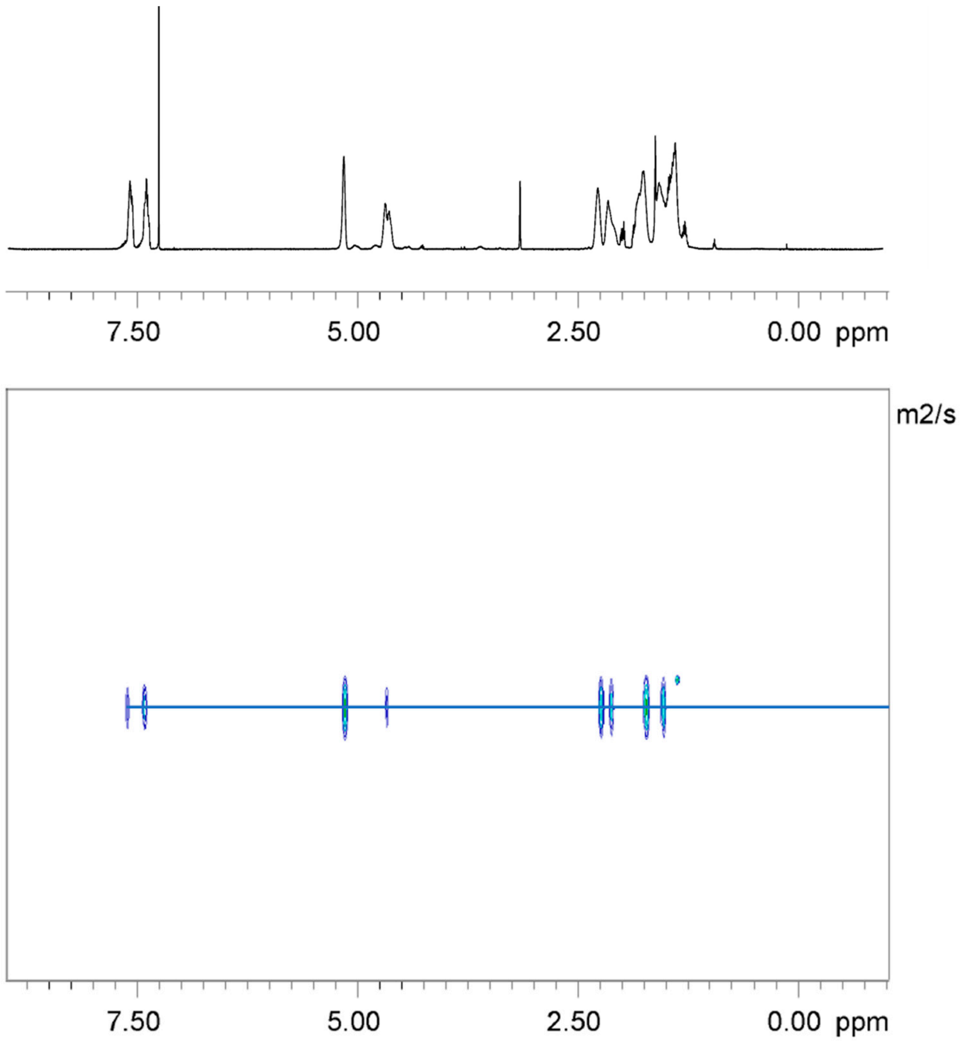

Figure S14. DOSY spectrum of poly(ester-*block*-carbonate) from mixture of PA, CHO and CO_2 in $CDCl_3$ (400 MHz, 298 K). Entry 1, Table 2.

Gel permeation chromatography analysis (GPC)

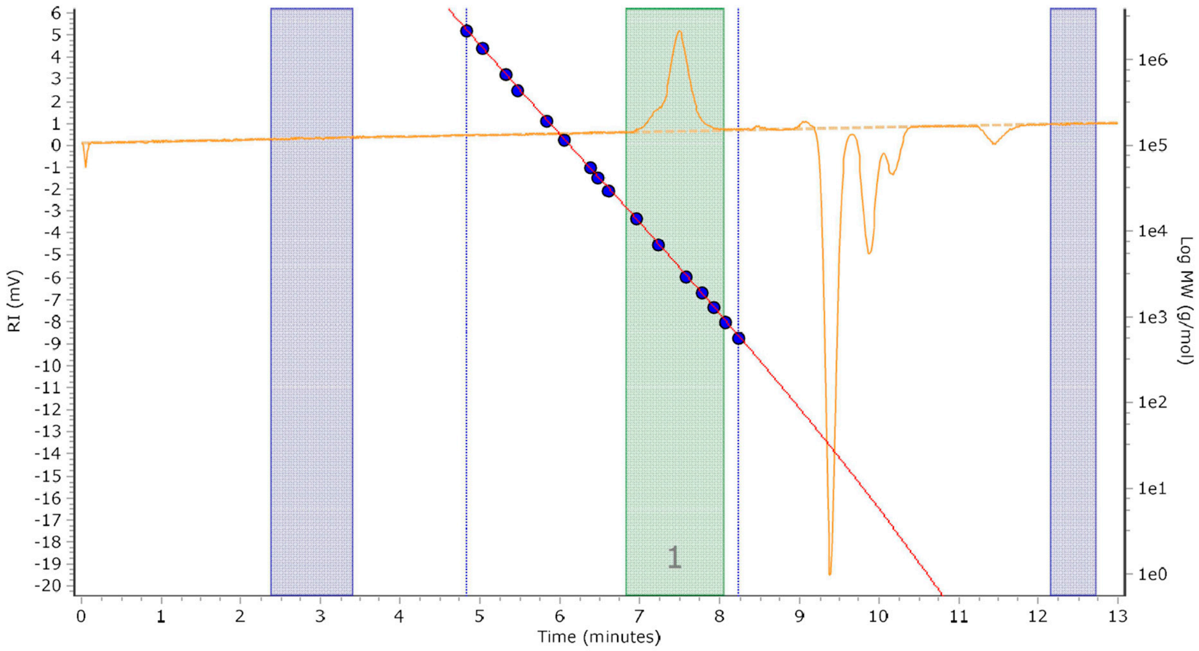

Figure S15. GPC trace of polyester poly(PA-*alt*-CHO). Entry 4, Table 2.

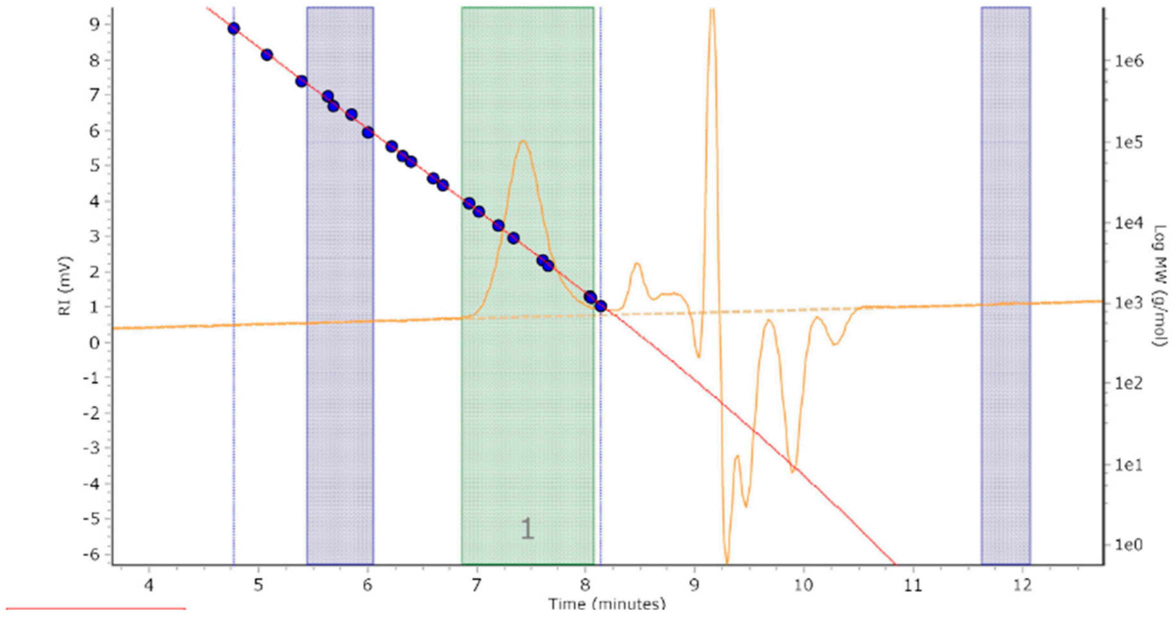

Figure S16. GPC trace of polyester poly(PA-*alt*-LO). Entry 6, Table 2.

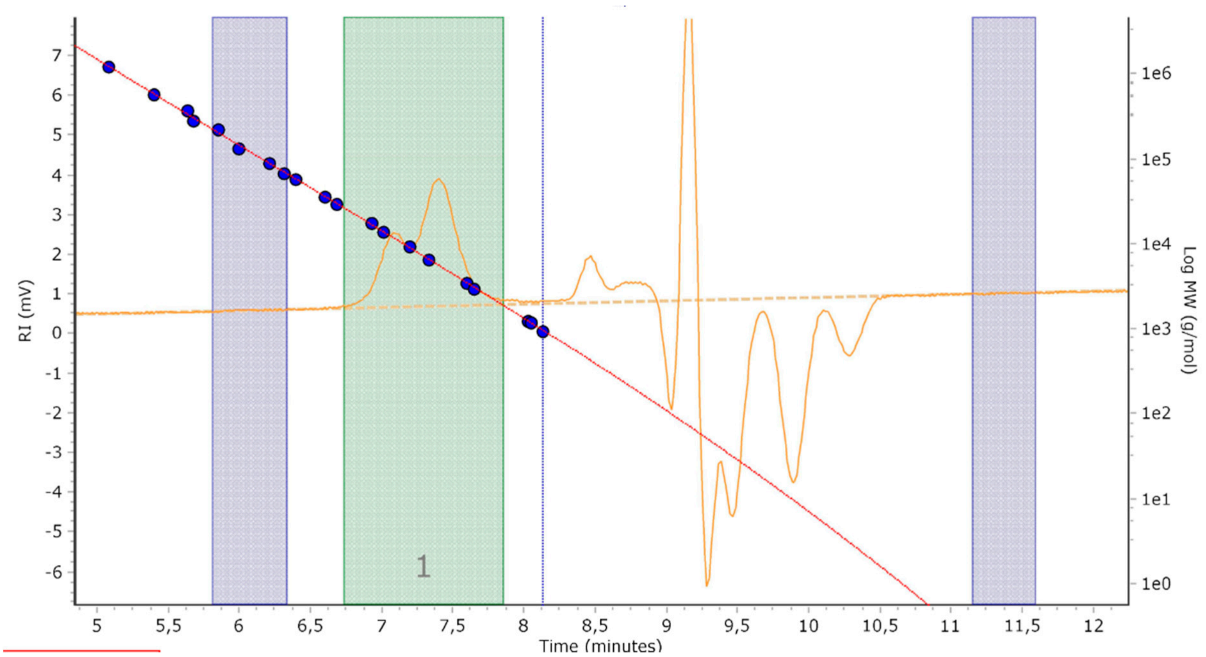

Figure S17. GPC trace of poly(cyclohexene carbonate). Entry 3, Table 1.

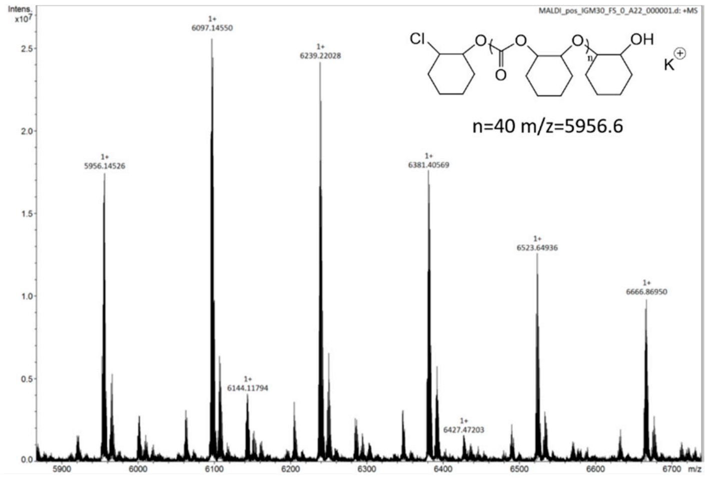

Figure S18. Expanded MALDI-ToF spectrum ranging from 5900 to 6700 of the PCHC obtained in entry 3 (Table 1) with the structure used for the assignment and the theoretical m/z value for 40 repeating units.

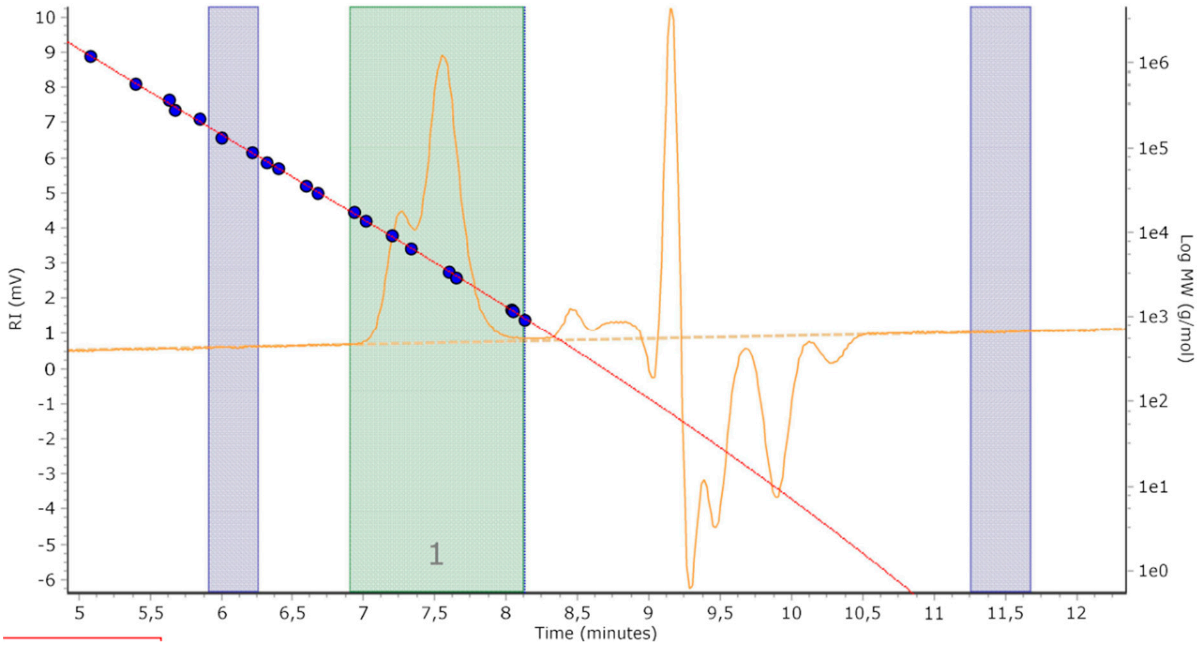

Figure S19. GPC trace of poly(ester-*block*-carbonate). Entry 2, Table 3.

Thermal gravimetric analysis (TGA)

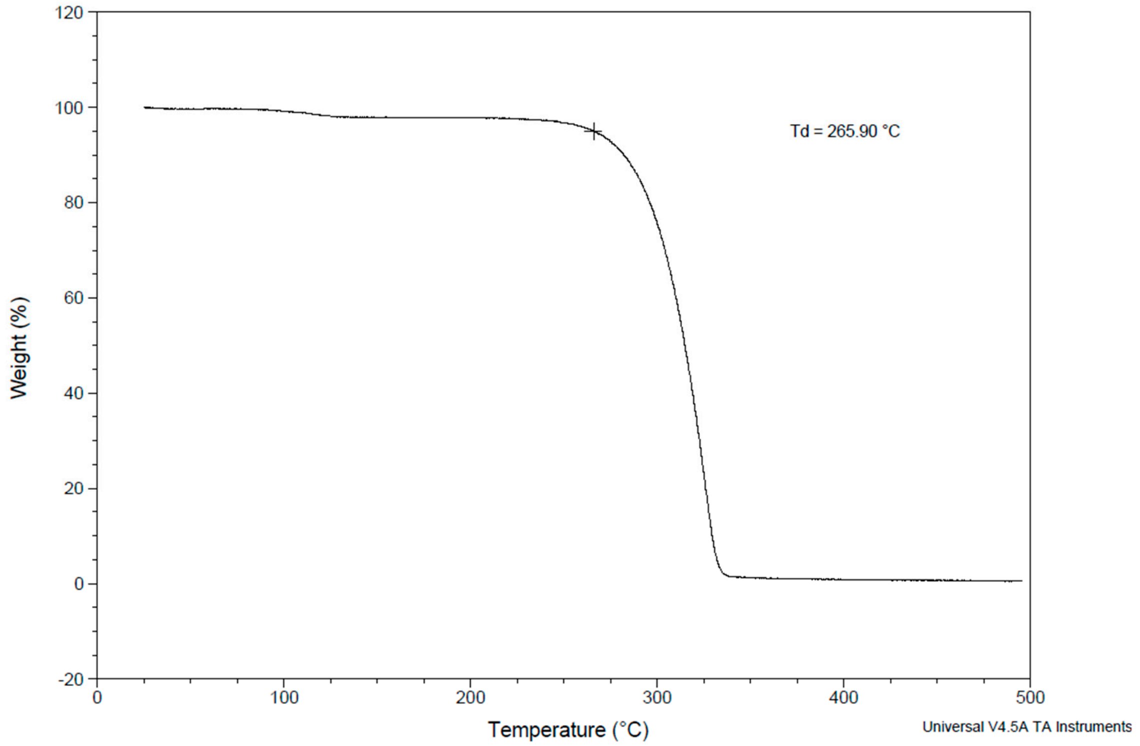

Figure S20. TGA of polyester poly(PA-*alt*-CHO). Entry 4, Table 2.

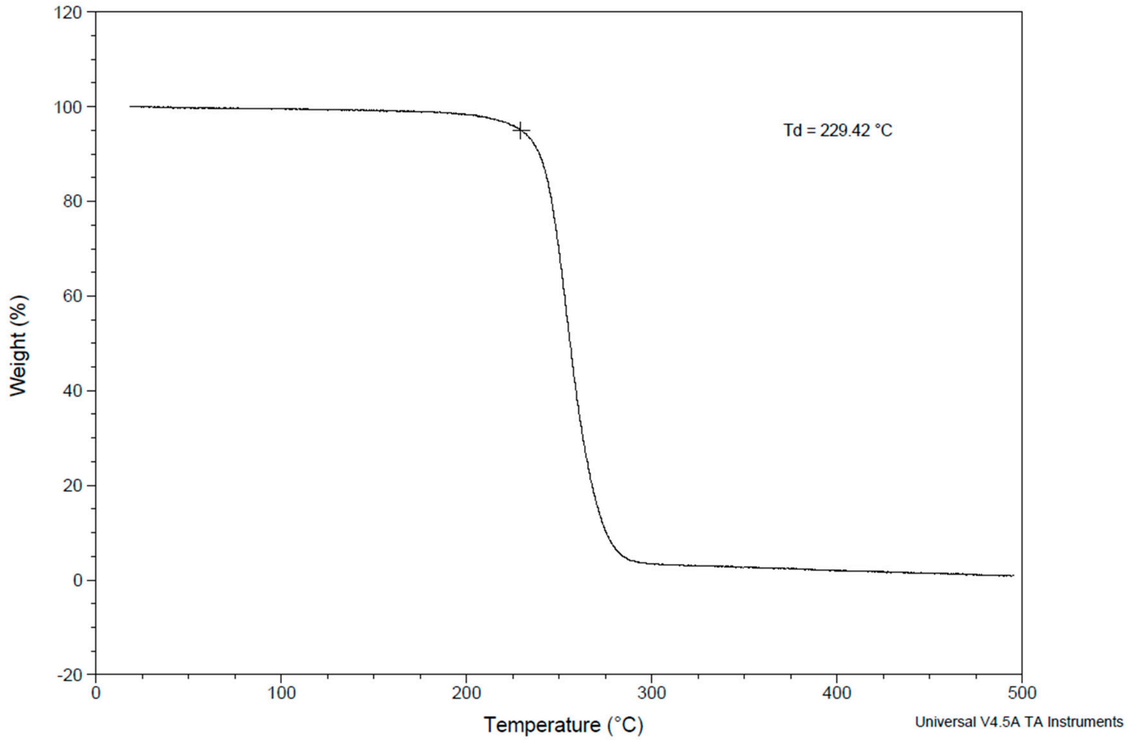

Figure S21. TGA of polyester poly(PA-*alt*-LO). Entry 5, Table 2.

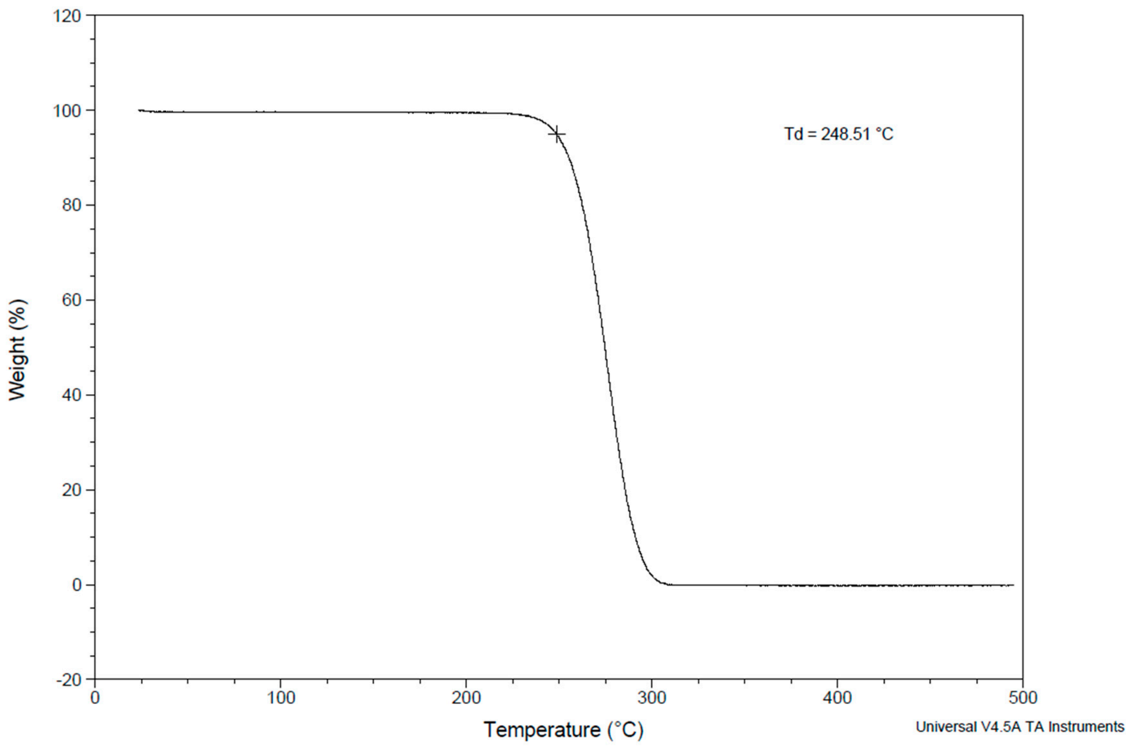

Figure S22. TGA of poly(cyclohexene carbonate). Entry 1, Table 1.

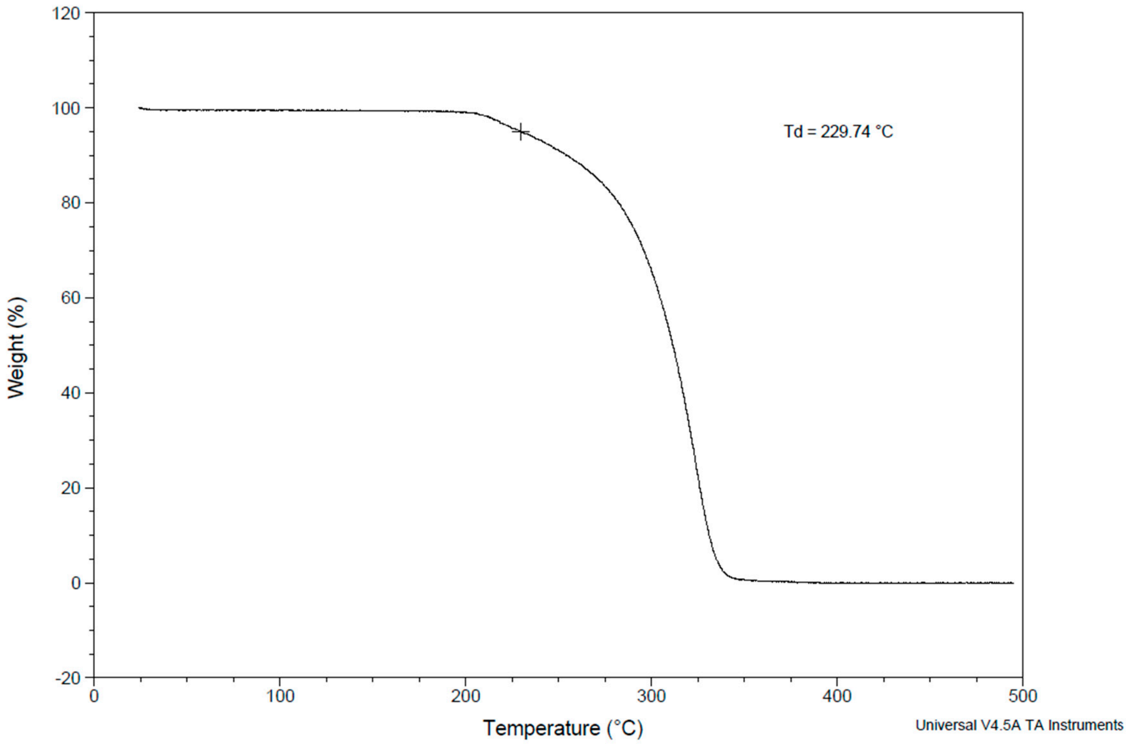

Figure S23. TGA of poly(ester-*block*-carbonate). Entry 3, Table 3.

Differential scanning calorimetry (DSC)

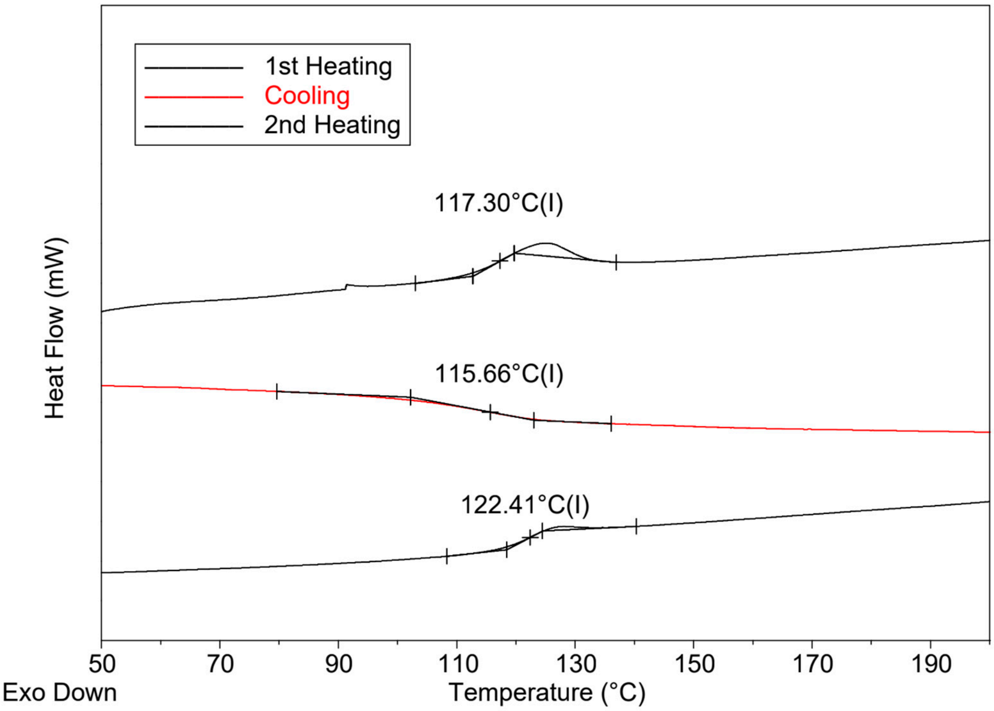

Figure S24. DSC of polyester poly(PA-*alt*-CHO). Entry 4, Table 2.

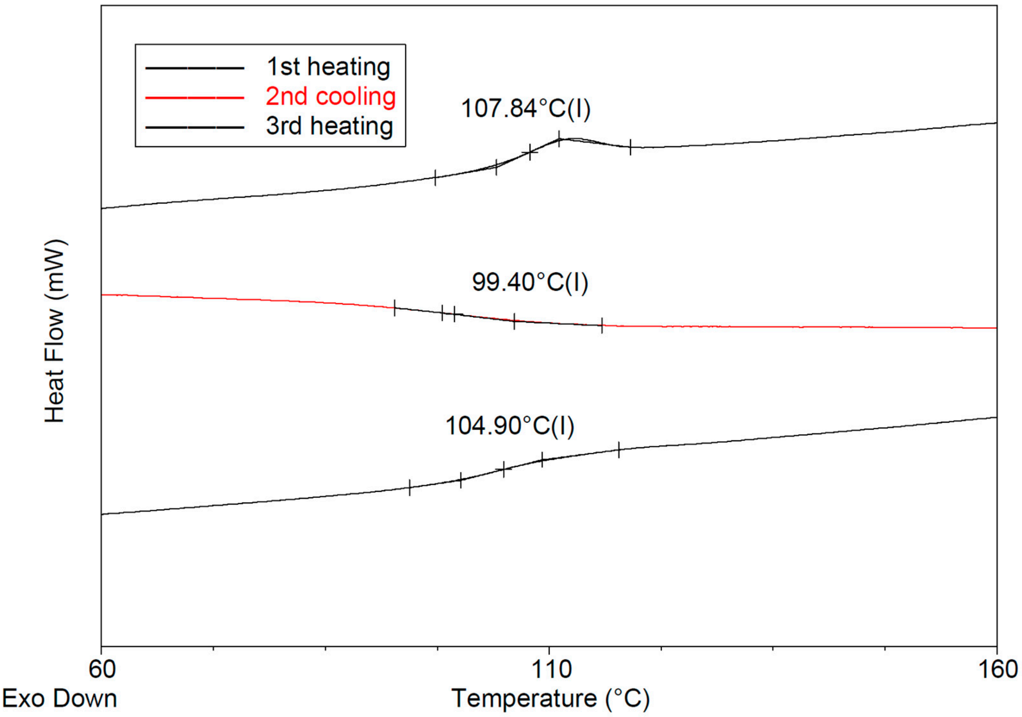

Figure S25. DSC of polyester poly(PA-*alt*-LO). Entry 5, Table 2.

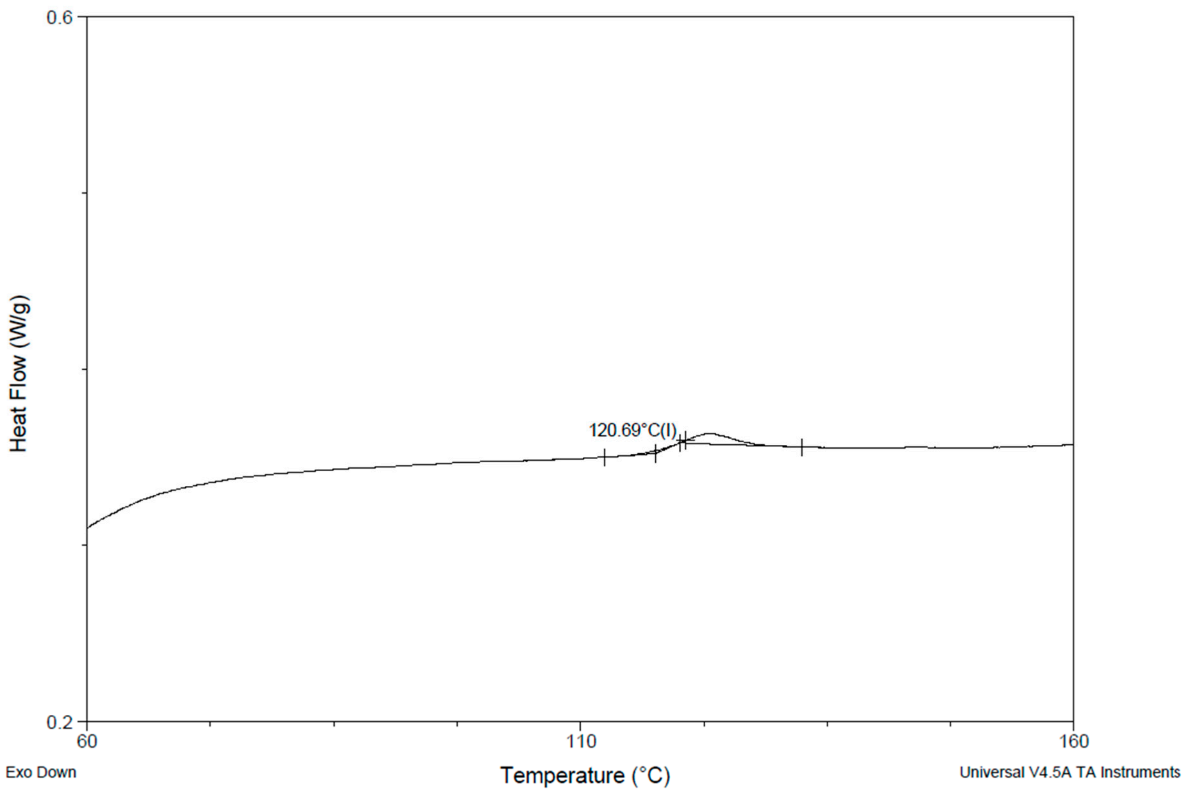

Figure S26. DSC of poly(cyclohexene carbonate). Entry 4, Table 2.

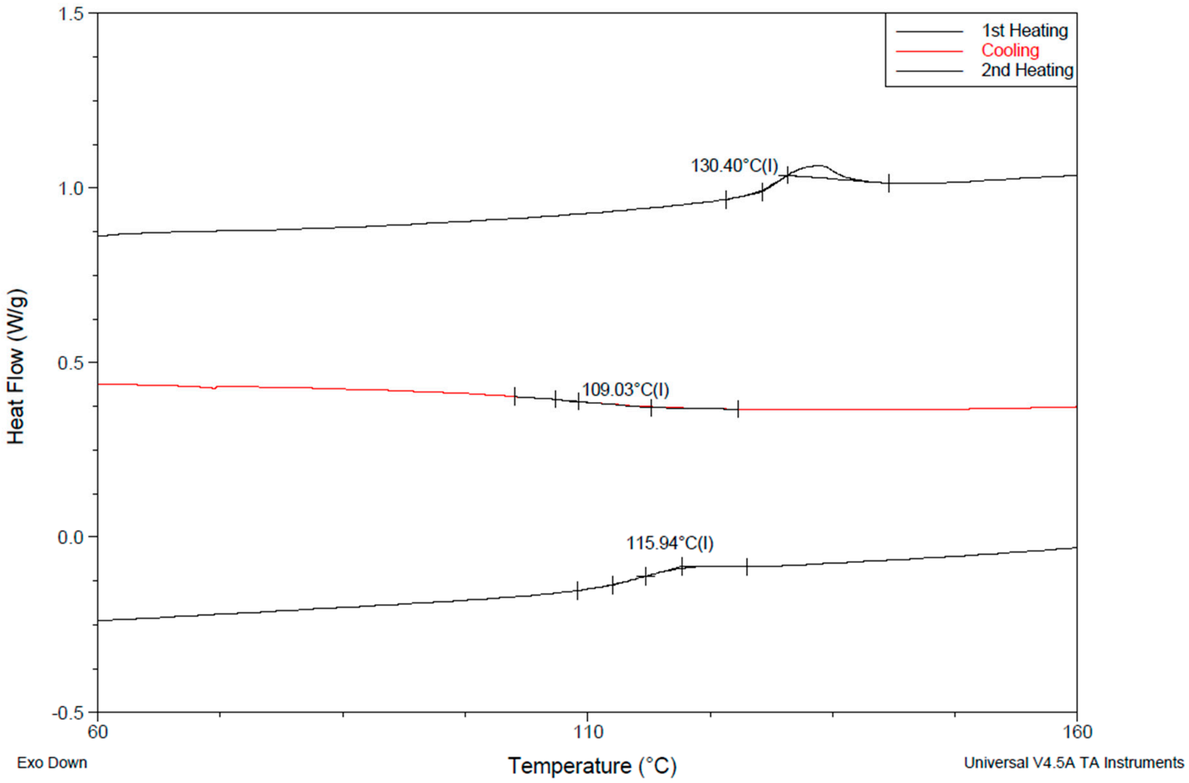

Figure S27. DSC of poly(ester-*block*-carbonate). Entry 1, Table 3.

5 References

[1] Li, B., Wu, G. P., Ren, W. M., Wang, Y. M., Rao, D. Y., Lu, X. B. *J. Polym. Sci, Part A: Polym. Chem*. **2008**, 46, 6102–6113.
[2] Yu, Y., Gao, B., Liu, Y., Lu, X.-B. *Angew. Chem. Int. Ed*. **2022**, 61, e202204492.
